# Cognition in anxious children with attention deficit hyperactivity disorder: a comparison with clinical and normal children

**DOI:** 10.1186/1744-9081-3-4

**Published:** 2007-01-15

**Authors:** Katharina Manassis, Rosemary Tannock, Arlene Young, Shonna Francis-John

**Affiliations:** 1 Department of Psychiatry, Hospital for Sick Children, 555 University Avenue, Toronto, Ontario, M5G 1X8, Canada; 2 Department of Psychiatry, University of Toronto, Toronto, Ontario, Canada; 3 Department of Psychology, Simon Fraser University, Burnaby, British Columbia, Canada; 4 Department of Psychology, Peel District School Board, Toronto, Ontario, Canada

## Abstract

**Background:**

Cognition in children with anxiety disorders (ANX) and comorbid Attention Deficit Disorder (ADHD) has received little attention, potentially impacting clinical and academic interventions in this highly disabled group. This study examined several cognitive features relative to children with either pure condition and to normal controls.

**Methods:**

One hundred and eight children ages 8–12 and parents were diagnosed by semi-structured parent interview and teacher report as having: ANX (any anxiety disorder except OCD or PTSD; n = 52), ADHD (n = 21), or ANX + ADHD (n = 35). All completed measures of academic ability, emotional perception, and working memory. Clinical subjects were compared to 35 normal controls from local schools.

**Results:**

Groups did not differ significantly on age, gender, or estimated IQ. On analyses of variance, groups differed on academic functioning (Wide Range Achievement Test, p < .001), perception of emotion (auditory perception of anger, p < .05), and working memory (backwards digits, p < .01; backwards finger windows, p < .05; Chipasat task, p < .001). ANX + ADHD and children with ADHD did poorly relative to controls on all differentiating measures except auditory perception of anger, where ANX + ADHD showed less sensitivity than children with ANX or with ADHD.

**Conclusion:**

Though requiring replication, findings suggest that ANX + ADHD relates to greater cognitive and academic vulnerability than ANX, but may relate to reduced perception of anger.

## Background

Anxiety Disorders (ANX) and Attention Deficit Hyperactivity Disorder (ADHD) both constitute major mental health problems of childhood, with affected children facing impairment at home, at school, and with peers [1]. Rates of ANX in the presence of ADHD range from 13 to 50% in various studies [2] and these comorbid children are less responsive to certain treatments [3] and are at even greater risk of long-term impairment and development of further psychopathology [4] than children with either "pure" condition. Although family studies suggest that the comorbid condition (ANX + ADHD) is more closely linked to anxiety disorders than ADHD [2], the best treatment for it is unknown.

To develop effective treatments and academic interventions for these children, an understanding of the cognitive processes that underlie ANX + ADHD is crucial. Effective treatments for anxiety, for example, place high demands on certain language-based cognitive processes [5]. Cognitive measures also provide a useful approach to investigating whether or not ADHD + ANX constitutes a unique subtype (ie. differs from either ADHD or ANX), since they constitute direct and objective measures and are independent of diagnostic criteria. For example, cognitive resemblance to either pure condition would suggest ANX + ADHD may have a similar etiology to that condition. Thus, cognitive studies have important therapeutic and etiological implications.

Studies of information processing are challenging to conduct for both theoretical and methodological reasons (reviewed in [6]). Small sample sizes and lack of measurement reliability, construct validity, or ecological validity are common methodological challenges. Use of stimulus sets inappropriate to age or developmental level and lack of attention to task-related fatigue are further challenges in child studies. Common theoretical challenges include insufficient construct specificity, examining cognitive processes in isolation (without reference to other aspects of information processing), and interpreting cognitive processes as contributing to psychopathology when they may be mere epiphenomena of clinical problems. Of course, it is also erroneous to assume that information processing factors in children always operate as they do in adults.

With these considerations in mind, we undertook an initial cognitive study and demonstrated that children with ANX + ADHD are cognitively distinct from either pure condition [7]. We examined several aspects of information processing, utilizing state-of-the-art measures of each construct, with age-appropriate stimuli, and a protocol that minimized order effects and subject fatigue. Consistent with high rates of comorbidity among certain anxiety disorders in this age group [8], ANX was defined as any anxiety disorder other than OCD or PTSD. We found that ANX + ADHD was associated with neither the inhibitory control deficits of 'pure' ADHD, nor the heightened sensitivity to negative emotions of 'pure' ANX. It is the only study to date to examine emotional perception in this population.

The present study sought to build on this work by further elucidating the cognitive characteristics of these children, in comparison to children with ANX only, children with ADHD only, and normal controls. Executive dysfunction, particularly impairments in inhibitory control and working memory, have been demonstrated repeatedly in ADHD (reviewed in [4]). Given that the foremost evidence-based treatment for anxiety disorders is cognitively based (cognitive-behavioral therapy; [5]), however, a 'pure' ANX comparison group was considered particularly relevant.

Cognition in anxious children has been characterized as biased by selective perception and selective memory for threatening stimuli or emotions (reviewed in [7]). Anxiety-related deficits in executive function, particularly working memory, have also been proposed [9]. There is some evidence to suggest that these may play a role in combined ANX and ADHD [10-12]. Therefore, we were particularly interested in examining emotional perception and working memory in children with ANX + ADHD relative to children with either 'pure' condition and to normal controls. This study represents a first step to further our etiological and therapeutic understanding of ANX+ADHD. The cognitive domains of interest are described briefly before detailing our hypotheses and methods.

### Emotional perception

The cognitive vulnerability to anxiety is thought to relate to an automatic tendency for anxious individuals to selectively encode emotionally threatening information (reviewed in [13]). This encoding bias is demonstrated when subjects show selective attention to threatening stimuli [13], selective interpretation of ambiguous stimuli as threatening [14], selective memory for threatening words [14] or interference with task performance due to exposure to threatening stimuli [15]. The bias is most evident on tasks involving *implicit *rather than explicit attention to threat [13], suggesting that a conscious decision-making process is not involved.

In children with clinical anxiety disorders, Vasey and others [16] demonstrated an attentional bias towards emotionally threatening words on a probe detection task. On a dichotic listening task, clinically anxious children showed enhanced perception of emotions relative to normal controls, possibly reflecting hypervigilance to emotional cues [17]. High trait anxious children have been found to show a bias toward negative relative to neutral information for conceptual memory tasks [18], a processing bias for generally threatening information on an emotional Stroop task [19], and a tendency to interpret homophones as threatening rather than neutral [20]. In the only study to date of emotional perception in anxious children comorbid for ADHD, these children showed reduced perception of emotions relative to normal controls [7], rather than the heightened perception of threatening or negative emotion characteristic of anxious children. On the other hand, the few available studies of emotional perception in ADHD yield inconsistent findings [21,22]. The findings also suggest that comorbid conduct problems may influence response to emotional stimuli [22,23].

### Working memory

Working memory is an aspect of executive function. It has been described as a limited capacity system for the temporary storage and processing of information [24]. It is thought to underlie a wide range of cognitive processes, including planning, learning, problem solving, reasoning and comprehension [25], and predicts academic achievement [26]. Tasks requiring working memory test the ability to simultaneously store and manipulate retained information in order to complete complex tasks [27]. For example, asking a subject to remember a particular number (storage) while adding a different set of numbers (manipulation) would constitute a test of working memory. Deficits in this ability are seen in a variety of clinical conditions.

In children with ANX, deficits may occur due to the interference of worry with normal processing of information [9]. Eysenck postulates that anxious individuals have clusters of anxiety-related information in long term memory which are easily accessible, rapidly activated, and retrieved quickly thus interfering with information-processing. Studies support an inability of anxious individuals to inhibit anxiety-provoking stimuli [28-30]. Anxiety enhances motivation, however, potentially compensating for the effect of worry on some tasks. Francis-John and colleagues (submitted) recently found impairment in complex verbal working memory in anxious children relative to normal controls, but not in visual-spatial working memory. However, the sample included a substantial number of children with comorbid ADHD. Working memory has not been examined in other studies of anxious children.

In ADHD, working memory impairments have been linked theoretically [31] to the disorder, and recent meta-analyses have empirically shown that working memory tasks discriminate between ADHD and controls [32]. Furthermore, these meta-analyses demonstrate impairments in visual-spatial as well as auditory-verbal working memory abilities in ADHD which remain robust even after controlling for comorbidity with other psychiatric disorders and general intellectual function [32].

Studies using complex tasks that place a high demand on working memory have shown greater impairment for children with internalizing disorders and ADHD than for those with ADHD alone [10-12]. Tannock et al. [12], using a serial addition task, showed that ANX + ADHD was associated with significantly worse performance on the slower trials of the task, but no difference at higher speeds where performance declined for children with ADHD. Compared to children in the ADHD-only group, those with ANX + ADHD also failed to show improvements on auditory-verbal working memory tasks when treated with stimulant medication [10]. Pliszka [10] administered a memory scanning task that placed demands on nonverbal working memory to children with ADHD with or without comorbid ANX. ANX + ADHD was associated with longer, sluggish reaction times on this task.

### Hypotheses

Based on the above, we hypothesized (1) that children with ANX would show heightened perception of negative emotions relative to the other children studied, a bias that might not be evident in the comorbid group [7]; (2) that children with ANX + ADHD would be particularly impaired on working memory relative to the other children studied, given that both anxiety and ADHD have been linked to working memory problems through different mechanisms.

We were also interested in overall academic ability, and hypothesized difficulty in all clinical groups relative to normal controls, but perhaps more so in the comorbid group.

## Methods

### Subjects

Children were recruited from two outpatient clinics (one specializing in anxiety disorders; the other in learning difficulties) in two university-based clinical research centers serving a large urban and suburban population. Investigators met regularly to ensure standardized procedures at the two clinics. A consecutive sample of children ages 8 to 12 years meeting study criteria were recruited. All racial and socioeconomic groups were represented, but with some over-representation of Caucasian versus non-Caucasian families and more affluent versus less affluent families, relative to the local census population.

Control subjects were recruited from local schools in the same geographic area, based on principals' nomination of students with no evidence of emotional or behavioral problems. Each student was also asked to nominate a friend (snowball technique). Controls were only included if they were within one standard deviation of the population mean for total scores on standardized measures of anxiety and ADHD symptoms (see below). While two potential controls were excluded for this reason, neither showed evidence of clinical disorder on interview.

All children were in an unmedicated state at the time of the study. Children receiving psychoactive medication (all had been diagnosed with ADHD and were on psychostimulants: 9 comorbid children and 6 ADD children) discontinued it for a period of seven medication half-lives prior to study participation, to ensure adequate washout. Children who had received a course of cognitive behavioral therapy (8 sessions or more) for their anxieties were excluded, as this form of therapy may alter cognitive processes. There were no differences among clinical groups for the number of mental health services received in the previous 4 years, nor for the proportion of children receiving ongoing mental health follow-up in the previous year (about 60% in each clinical group).

Prior to participation, potential subjects and their parents completed the Anxiety Disorders Interview Schedule (ADIS; [33]), a well-validated, semi-structured diagnostic interview using DSM – IV criteria. Diagnosis was based primarily on this instrument, but teacher reports (by structured telephone interview and Conners' Questionnaire[34]) were required to confirm ADHD in the school environment. Anxiety disorders were diagnosed when present either by parent or child report. All interviewers were child psychiatrists or child psychologists, trained to reliability on the instrument, and with at least 3 years experience using it in other research studies. With parental consent, 10% of these diagnostic interviews (randomly selected) were videotaped and scored by an independent rater to ensure reliability among interviewers. No discrepancies between interviewers and raters were found for group assignment. Children also completed the Multidimensional Anxiety Scale for Children (MASC; [35]) and parents the Parent Conners' Questionnaire [34], to obtain well-validated, continuous measures of anxiety and ADHD symptoms respectively.

All ADHD subtypes were included, and all childhood anxiety disorders were included apart from post-traumatic stress disorder and obsessive compulsive disorder (as the latter are likely to be cognitively distinct). Childhood anxiety disorders are highly comorbid: 40% of anxious children have more than one disorder [8], so mixed anxious samples are commonly studied in this type of research. Generalized Anxiety Disoder (GAD) was the primary diagnosis in about 50% of both the ANX and ANX + ADHD groups.

Because this was a clinical sample, other comorbid conditions (Learning Disabilities, Oppositional Defiant Disorder, Conduct Disorder, Depression) were expected. Although these could potentially confound interpretation of data, they are so frequent in this population that excluding subjects with them would likely have rendered the study non-feasible. There is no evidence that comorbid Oppositional Defiant Disorder alters the cognitive features of ANX or ADHD. The other three comorbidities listed were assessed for each participating child. LD was defined as a standardized score on the Wide Range Achievement Test (WRAT-R; [36]) that was at least 1.5 SD below mean for age and below full-scale IQ score on the WISC-III [37]. Depression was defined by a score > 16 on the Children's Depression Inventory [38,39], or by the diagnostic interview. CD was assessed by the diagnostic interview. In practice, there were no children meeting diagnostic criteria for either depression or CD. There were several children in each clinical group meeting criteria for LD by the above definition (39 total). However, chi-square analyses showed significant group differences for reading disability only (6 anxious, 3 comorbid, and 6 ADHD children; chi-square = 12.10, p = .007). Post hoc analyses used continuous measures of comorbidity as covariates in interpreting data, to detect any comorbidity-related effects (analysis described in [40]).

We excluded children who had a full-scale IQ < 80, lacked fluency in English, or were suffering from psychosis or serious visual, auditory, or speech deficits.

### Procedure

The project was approved by our hospital Research Ethics Board, and children and parents provided informed assent and consent respectively. Research was carried out in compliance with the Helsinki Declaration. Administration of measures was done by a research technician blind to child diagnosis, and counterbalanced to control for any order effects. Children completed the Wide Range Achievement Test-Revised (WRAT-R, [36]; tests academic achievement) and the Vocabulary and Block Design subtests of the Wechsler Intelligence Scale for Children (WISC-III [41]). This two-subtest short form has been shown to reliably estimate verbal and non-verbal intelligence respectively [42]. Parents were asked to complete a set of questionnaires and to provide basic demographic information (Ontario Child Health Study, [43]). Normal hearing was confirmed by audiological screening and handedness was verified by a preference inventory (Waterloo Handedness Questionnaire, [44]). If the child had completed either a WRAT-R or WISC-III within the past year, scores were obtained with parental consent and the test was not re-administered.

### Cognitive measures

Measures targeted the cognitive domains of interest, with verbal and nonverbal working memory examined separately. A timed verbal working memory task was also included, as the stress of a timed task may affect anxious children differently than non-anxious children. All measures are widely used research tools, and have acceptable reliability and validity data.

### Emotional perception

Diagnostic analysis of nonverbal accuracy 2 (DANVA2)[45]: The DANVA2 consists of four receptive and three expressive subtests designed to measure the accuracy of nonverbal social information processing in children. Two receptive subtests (Adult Faces 2 and Adult Voices 2) were selected for use in the current study. Children had to identify the emotional valence of the facial expression or tone of voice respectively. Adult stimuli were chosen because child stimuli on the measure are less subtle, sometimes resulting in ceiling effects.

### Working memory: verbal

CHIPASAT (Children's paced auditory serial addition test; [46]): The CHIPASAT is a precisely timed task in which tape-recorded, single-digit numbers are presented in trials of differing speeds. Children are required to add each new number to the immediately preceding number and give the answer aloud. The numerical knowledge required is usually acquired by grade 1. The traditional dependent variable is the total number of correct responses for each speed. Some strategies for doing the task (e.g., adding stimuli discontinuously; [12]) allow for many correct responses without a high demand on working memory, so variables pertaining to strategy were also compared across groups. These were found not to differ by group, however, so correct responses only are reported in this paper. More detailed analyses of this task for a subset of this sample can be found in Francis-John et al., (submitted).

Backward digit span (WISC-III;[41]): Standard procedure for the WISC-III was used, and the highest number of digits recalled under the Backward condition was recorded (whether or not the child was able to recall the same number of digits once or twice), then converted to a scaled score. Only scaled scores were reported and used in analyses.

### Working memory: nonverbal

Finger windows backwards: The Finger Windows subtest from the Wide Range Assessment of Memory and Learning (WRAML) [47] was administered. In the forward administration, the examiner indicates a series of spatial locations by inserting a pencil through a series of randomly spaced holes ("windows") on an 8 × 11 inch card at a rate of one hole per second. The child must then reproduce the same visual-spatial sequence by putting his or her finger through each window in the same order as the presentation. Items gradually increase in length from sets of 2 to sets of 6 windows, demonstrating spatial memory span. With backward administration, that is the child reproducing the sequences in backward order, this becomes a measure of spatial working memory. Lower scores are associated with greater impairment in this ability. Standard scores were used for the forward administration, and raw scores for the backward version.

### Statistical analysis

The Statistical Package for the Social Sciences – PC version (SPSSPC) was used for all analyses. Groups were compared on demographic characteristics and cognitive characteristics using analyses of variance (ANOVA). Chi-square was used for categorical measures. To test our hypotheses about specific group differences, post-hoc tests were done for all variables that showed significant group differences on ANOVA, with Bonferroni correction for multiple comparisons.

To further corroborate relationships between ADHD, ANX, and various cognitive measures, bivariate correlations between these measures and maternal Conners' ADHD Symptom Index and child MASC scores, respectively, were examined in secondary analyses. Further post hoc analyses were done to include estimated IQ (based on Vocabulary and Block Design scores) and each of the three comorbidities likely to affect cognition (LD, Depression, CD) measured as continuous variables. These variables were entered as covariates, one at a time, in analyses of covariance (ANCOVAs) to determine if between-group differences remained, as described by Nigg et al. [40]. Thus, we determined the effect (if any) of these comorbid conditions on our results.

## Results

Sample characteristics by group are described in Table 1. There were no significant group differences in age, gender, socioeconomic status, or handedness. As expected, ANX and ANX + ADHD groups reported significantly more anxiety than normal controls, although their mean scores still appeared to be in the normal range. This is not unusual in clinics where the anxiety prompting referral is identified by parents or clinicians (rather than the children themselves), given that correspondence between informants is only fair in childhood internalizing disorders [48]. On the Conners', mothers reported significant differences between children with ADHD, ANX, and normal controls, while teachers only distinguished clinical versus nonclinical groups.

**Table 1:** Description of Sample: Means and Standard Deviations^a^

	Anxiety Only (n = 52)	Comorbid for ADHD* (n = 35)	ADHD Only (n = 21)**	Normal Controls (n = 35)	Total (n = 143)
Age in Years	9.42 (1.23)	9.82 (1.27)	9.60 (1.43)	9.71 (1.32)	9.61 (1.28)
Socioeconomic Status (Hollingshead Index [54])	48.45 (12.21)	46.56 (15.46)	44.22 (18.36)	53.92 (8.85)	48.19 (13.61)
Gender (% Males)	66%	74%	81%	63%	69%
Handedness (% Right vs. Left/Mixed)	67%	53%	70%	74%	64%
DSM-IV Conners' ADHD Symptom Index (t-score) Mother	57.08 (11.09)	69.00 (12.63)	69.80 (7.00)	49.50 (7.67)^3^	58.86 (12.81)
Report Teacher Report (n = 69)	55.15 (12.35)	61.11 (18.81)	74.33 (11.68)	50.08 (5.87)^2^	56.01 (14.60)
Multidimensional Anxiety Scale for Children (t-score)	53.12 (1.08)	54.97 (13.24)	50.10 (11.00)	46.23(10.09)^2^	51.52 (11.82)

^a ^standard deviations are shown in brackets; ^1 ^p < .05, ^2 ^p < .01, ^3 ^p < .001

* 19 Inattentive Type; 16 Combined Type

** 11 Inattentive Type; 10 Combined Type

Data was screened for outliers and conformity with the statistical assumptions of analysis of variance. Also, the effect of specific anxiety diagnosis was examined by comparing children with a primary diagnosis of GAD (about 50% of both ANX and ANX + ADHD groups) with those with other primary anxiety diagnoses on all measures of interest. No significant differences were found, so all anxiety diagnoses were examined together in subsequent analyses.

To test our main hypotheses regarding emotional perception in ANX and working memory in ANX + ADHD, groups were compared on measures relevant to these domains. Table 2 shows group means on these cognitive measures of interest. Group differences were found on all academic achievement tests, the Chipasat (verbal working memory), Digit span backwards (verbal working memory), finger windows backwards (nonverbal working memory), and DANVA – adult angry voice (emotional perception). Group differences are reported uncorrected in the table. However, we also applied the Bonferroni correction within each domain, consistent with our initial hypotheses. Doing so, the following comparisons remained significant: all academic achievement differences, the Chipasat 2.0 and 2.8, and the Digit Span Backwards.

**Table 2:** Cognitive Tasks by Group

TASK	ANX	ANX + ADHD	ADHD	NORMAL	Signif. ANOVA	Effect Size (Partial Eta Squared)
WISC-III: IQ	107.1 (12.4)	103.53 (10.95)	103.37 (12.63)	110.41(8.42)	-	-
Perception of Emotion: DANVA						
Happy (raw)	3.79 (1.54)	4.21 (1.43)	3.37 (1.38)	4.09 (1.36)	-	-
Sad (raw)	4.17 (1.12)	3.85 (1.16)	3.84 (1.34)	3.83 (1.15)	-	-
Angry (raw)	4.58 (1.01)	4.03 (1.27)	4.79 (0.98)	4.26 (0.89)	*	.063
Fearful (raw)	4.08 (2.34)	3.71 (1.71)	3.95 (1.68)	3.83 (1.64)	-	-
Working Memory: Nonverbal: Finger – Windows						
Forward (scaled)	8.91 (3.46)	7.91 (2.94)	9.19 (2.82)	9.39 (2.91)	-	-
Backward (raw)	10.13 (4.78)	9.32 (4.23)	7.74 (5.20)	11.33 (3.79)	*	.059
Working Memory Verbal: Digit Span						
Forward (scaled)	10.81 (2.81)	10.90 (3.30)	9.00 (3.04)	10.63 (3.23)	-	-
Backward (scaled)	10.67 (2.74)	8.84 (3.50)	7.94 (3.17)	10.40 (3.25)	**	.099
Chipasat Task						
Speed: 2.8 (raw)	31.49 (12.21)	25.60 (8.43)	23.41 (11.02)	34.88 (9.76)	***	.101
Speed: 2.0 (raw)	25.51 (10.65)	22.00 (7.62)	18.82 (7.68)	29.36 (7.55)	***	.084
Academics WRAT:						
Reading	110.4 (14.6)	105.41 (17.59)	99.71 (19.20)	118.63 (11.04)	***	.144
Spelling	106.7 (51.1)	96.94 (15.51)	93.52 (17.17)	113.09 (11.85)	***	.193
Arithmetic	99.3 (15.5)	92.27 (15.99)	93.05 (16.48)	109.89 (10.59)	***	.176
Word attack	108.4 (13.8)	100.12 (16.03)	94.05 (20.93)	109.12 (8.13)	***	.131

*p < .05 **p < .01 ***p < .001; t-scores unless otherwise indicated;

WRAT, Chipasat, and Digit Span Backward significant after Bonferroni correction;

WISC-III = Wechsler Intelligence Scale for Children-III; WRAT = Wide Range Achievement Test; DANVA = Diagnostic Assessment of Nonverbal Accuracy

For all significant ANOVAs, post-hoc analyses were done to test our hypotheses regarding specific group differences (see Table 3). As hypothesized for emotional perception, ANX were indeed more sensitive to adult anger (the most threatening emotion) than ANX + ADHD. Interestingly, children with ADHD also showed this sensitivity relative to ANX + ADHD. For working memory (second hypothesis), both ADHD groups appeared impaired relative to normal controls, but ANX did not. The effect appeared somewhat stronger for verbal working memory, with ANX + ADHD showing only a trend level difference from normal controls on nonverbal working memory. Significant academic impairments were evident in all clinical groups relative to normal controls, but appeared more pronounced in the ADHD groups.

**Table 3:** Post-hoc Group Comparisons for Differentiating Measures (p values; in bold if significant)

Measure	ANX vs. ANX + ADHD	ANX vs. ADHD	ANX vs. Normal	ANX + ADHD vs. ADHD	ANX + ADHD vs. Normal	ADHD vs. Normal
DANVA angry	**.026**	.447	.121	**.028**	.389	**.047**
Chipasat (2.8)	**.037**	**.021**	.233	.470	**.001**	**.001**
Chipasat (2.0)	.162	**.023**	.119	.198	**.001**	**.000**
Digits Backward	**.009**	**.001**	.674	.384	.064	**.013**
Finger Windows Backward	.423	.071	.225	.234	**.045**	**.006**
WRAT reading	.158	**.012**	**.006**	.265	**.000**	**.000**
WRAT spelling	**.005**	**.002**	**.037**	.453	**.000**	**.000**
WRAT arith.	**.046**	.128	**.001**	.864	**.000**	**.000**
WRAT w. attack	**.012**	**.001**	.778	.236	**.005**	**.000**

### Secondary analyses

To determine whether anxiety and ADHD measured as continuous variables related to cognitive measures regardless of diagnostic group, bivariate correlations were examined for the whole sample between Mother Conners' DSM-IV ADHD Symptom Index and the cognitive measures of interest. Significant correlations (p < .05) were evident for: Chipasat 2.0, Chipasat 2.8, Digit Span Backwards (ie. verbal working memory measures), WRAT Spelling, and WRAT Arithmetic subtests. All correlations were in the expected direction (ie. worsening test performance with increasing Mother Conners' symptoms). Correlations were also examined between the child-report MASC (our continuous measure of anxiety) and the cognitive measures of interest. A significant correlation (p < .05) was evident for DANVA: angry adult voice, detected more by children with higher MASC scores.

When covarying for estimated IQ, all previously significant comparisons remained significant. When covarying for depressive symptoms and for conduct disorder symptoms, all previously significant comparisons remained significant. To assess the effect of learning problems, we ran group comparisons covarying for the discrepancy between estimated IQ and specific WRAT scores for each subject, consistent with our operational definition of LD above. Using this approach, all previously significant working memory and emotional perception comparisons remained significant (obviously not applicable to WRAT comparisons). Interestingly, covarying for the actual WRAT scores (without relating them to IQ) showed that reading ability on WRAT eliminated all group differences apart from that for the DANVA: auditory anger.

Although we did not hypothesize any gender differences, we re-ran all group comparisons covarying for gender. All significant group differences remained, and a significant effect for gender was found only on one measure, the Chipasat, where females tended to score lower than males at the slower speed (F = 10.66, p < .01).

## Discussion

Although effect sizes were modest, group differences between anxious children, anxious children comorbid for ADHD, and normal controls were found on several cognitive measures. The lack of group differences in demographic characteristics (age, gender, socioeconomic status) suggests that these factors are unlikely to account for these findings. The degree of anxiety reported for the two ANX groups was also very similar, as was the degree of severity of ADHD symptoms in the two ADHD groups, suggesting that cognitive differences are unlikely to be due to symptom severity in one or the other group. Clinical groups were also comparable in prior mental health service utilization, suggesting that length of illness is unlikely to account for cognitive differences.

### Specific findings

We hypothesized that 'pure' anxious children would show enhanced perception of negative emotions. Consistent with this hypothesis, 'pure' anxious children were found to have an enhanced perception of auditory anger relative to comorbid children, with normal control scores intermediate between these two groups. This finding partially replicates our previous work showing reduced perception of negative emotion in ANX + ADHD [7]. Our continuous measure of anxiety (MASC) was also correlated with perception of auditory anger. Anger is considered the most threatening of negative emotions (from the listener's perspective) so it was not surprising that sensitivity to it was heightened in ANX, where threat sensitivity has been confirmed in other studies [7,16]. Notably, the ANX + ADHD group was least sensitive to this emotion, suggesting that their anxiety may be of a different nature to that of the ANX group and that anxiety may not relate to threat sensitivity. If replicated, this finding suggests that other etiological mechanisms for anxiety should be examined in this population (see below). Interestingly, 'pure' ADHD children also showed the perceptual bias towards anger relative to ANX + ADHD.

In relation to working memory, we hypothesized the greatest impairment in ANX + ADHD. Instead, we found that both ADHD groups were impaired relative to normal controls and ANX. This difference was more robust for verbal than nonverbal working memory, and occurred whether or not the task was timed. Maternal (continuous) report of ADHD symptoms also correlated with verbal working memory. These findings are consistent with previous reports suggesting that lack of vigilance or distraction by external stimuli can interfere with working memory in ADHD [49]. We did not replicate earlier findings of greater impairment in ANX + ADHD than in 'pure' ADHD [10-12]. The findings appear contrary to Eysenck and Calvo's [9] hypothesis regarding the adverse effects of worry on working memory. It is possible, however, that findings in ANX might be different in a less reassuring test situation than that in our laboratory. Studies of such children in anxious states (for example, during examinations at school) might show greater effects of worry on working memory.

Academic differences were greatest between children with ADHD and normal controls, though some academic impairment was evident in ANX as well. ANX + ADHD did not appear to have worse academic performance than children with 'pure' ADHD, highlighting the need for school supports for all children meeting ADHD criteria.

We examined other reasons for the group differences (apart from diagnosis per se) by covarying for IQ, gender, academic deficits relative to IQ, comorbid depressive symptoms, and comorbid conduct symptoms. All group differences remained significant, and only one measure (Chipasat at slower speed) showed a significant gender effect (males performed better). Controlling for academic ability, however, eliminated all but one group difference: the difference in auditory perception of anger. Thus, emotional perception may be relatively independent of academic ability, but working memory appears to show some association. This association disappears, however, when academic ability is measured relative to IQ. This finding suggests that group differences in working memory cannot be accounted for entirely by differential academic deficits.

### One possible model for ANX + ADHD

Although needing replication, these findings have potentially interesting implications for understanding the comorbidity between ANX and ADHD. Vance and Luk [50] hypothesized that a neurodevelopmental deficit underlies both anxious and ADHD symptoms in these children. Our findings suggest one candidate for such a deficit: impaired working memory, especially verbal working memory. Working memory has been linked to the frontal lobes, with verbal ability predominantly on the left side in most individuals. Studies linking approach behavior in anxious individuals to left anterior activation on EEG [51], reduced left anterior activation in behaviorally inhibited children [52], successful treatment of anxiety using verbally mediated cognitive behavioral strategies [5], and deficits in frontal lobe functions in ADHD [53] are all consistent with this idea. The lack of sensitivity to threat (angry voice) we found in ANX + ADHD (in contrast to 'pure' ANX) is also consistent with this model. Thus, children with ADHD + ANX may have frontal lobe deficits that affect their ability to inhibit both negative affect (resulting in anxiety symptoms, even in the absence of threat sensitivity) and responses to stimuli (resulting in ADHD symptoms). Understanding the nature of these impairments more precisely may suggest fruitful avenues of clinical and academic intervention for them.

### Limitations

Further studies that include larger, more diverse samples are needed to replicate and extend these findings. The collection of the sample from two university-based clinics may also have resulted in some sample bias. This bias may limit generalizability to other populations (for example, children with ANX + ADHD in the community). Vasey and colleagues [6] have also advocated the use of multiple measures of each construct to allow the generation of composite scores, and longitudinal studies that clarify the relationship between deficit and disorder (eg. determining if deficits are present prior to the onset of disorder or can be modified such that symptomatology changes).

### Clinical implications

The nature of the cognitive deficits in ANX + ADHD may be relevant to these children's ability to benefit from cognitive behavioral therapy for anxiety, particularly since these deficits are less likely to be ameliorated by stimulant treatment than in 'pure' ADHD children. Thus, even a medicated child with comorbid ANX + ADHD may find the verbal reasoning required in cognitive behavioral therapy confusing, due to limited verbal working memory. ADHD may also make the child appear disruptive or difficult to manage if CBT is offered in a group format (increasingly favored to contain costs). CBT may require modification and/or administration in an individual format in order for these children to benefit.

Given the above deficits, children with ANX + ADHD are also likely to be disadvantaged academically. They may require different teaching strategies geared to their unique cognitive profile. If they encounter school failure, this may exacerbate anxiety leading to further problems. Enhancing teachers' and clinicians' awareness of the particular vulnerabilities of these children may improve their therapeutic and academic outcomes.

## Conclusion

Findings suggest that ANX + ADHD relates to greater cognitive and academic vulnerability than ANX, especially with respect to working memory, but may relate to reduced perception of anger. Awareness of this vulnerability may allow tailoring of psychological and academic intervention to better meet the needs of affected children. Further studies of larger, more diverse samples are indicated to replicate these findings and further elucidate the cognitive and neurological substrates of ANX + ADHD.

## Competing interests

The author(s) declare that they have no competing interests.

## Authors' contributions

KM was the principal investigator of this study, principal author of this paper, and leads one of the participating outpatient clinics. RT was a co-investigator of this study, and developed the initial study design jointly with KM. AY contributed valuable ideas to the final design and procedures of the study, and leads the second participating outpatient clinic. All authors read and approved the final manuscript.
